# Macrophage imaging and subset analysis using single-cell RNA sequencing

**DOI:** 10.7150/ntno.50185

**Published:** 2021-01-01

**Authors:** Sean Arlauckas, Nuri Oh, Ran Li, Ralph Weissleder, Miles A. Miller

**Affiliations:** 1 Center for Systems Biology, Massachusetts General Hospital Research Institute, Boston, MA 02114, USA; 2 Department of Radiology, Massachusetts General Hospital and Harvard Medical School, Boston, MA 02115, USA; 3 Department of Systems Biology, Harvard Medical School, Boston, MA 02115, USA

## Abstract

Macrophages have been associated with drug response and resistance in diverse settings, thus raising the possibility of using macrophage imaging as a companion diagnostic to inform personalized patient treatment strategies. Nanoparticle-based contrast agents are especially promising because they efficiently deliver fluorescent, magnetic, and/or radionuclide labels by leveraging the intrinsic capacity of macrophages to accumulate nanomaterials in their role as professional phagocytes. Unfortunately, current clinical imaging modalities are limited in their ability to quantify broad molecular programs that may explain (a) which particular cell subsets a given imaging agent is actually labeling, and (b) what mechanistic role those cells play in promoting drug response or resistance. Highly multiplexed single-cell approaches including single-cell RNA sequencing (scRNAseq) have emerged as resources to help answer these questions. In this review, we query recently published scRNAseq datasets to support companion macrophage imaging, with particular focus on using dextran-based nanoparticles to predict the action of anti-cancer nanotherapies and monoclonal antibodies.

## Introduction

Non-invasive quantification of macrophages (MΦ) by imaging has shown potential in applications where their heterogeneous behaviors contribute to disease progression and drug responses, including in infection, inflammation, and cancer. MΦ can be among the most abundant cell-types in local diseased tissues and can affect drug action through direct drug engagement and indirectly by altering multicellular signaling within tissue. Drug classes including biologics and nanomaterials are especially influenced by MΦ, which interact through MΦ-expressed complement, lipid, scavenger, and Fc receptors. MΦ also influence pharmacology without binding or transporting drugs directly, for instance through shaping vascular function and inflammatory signaling. These cells contribute to wound healing, angiogenesis, fibrosis, blood-brain-barrier function, and coagulation processes that broadly govern drug transport 1.

Therapeutics continue to be developed that target or are influenced by MΦ 2, and many of these agents exhibit high patient-to-patient variability in both safety and efficacy. This variability is particularly acute in the treatment of solid cancers. As one example, the liposomal irinotecan nanotherapy (marketed as Onivyde for the treatment of metastatic pancreatic adenocarcinoma) 3 has been developed in principle to deliver toxic chemotherapy payloads more selectively and safely to tumors via passive “enhanced permeability and retention” (EPR) effects 4, 5. While evidence for EPR effects are strong in some patients with highly vascularized tumors marked by dysfunctional lymphatics and phagocyte infiltration, in other cases tumors are fibrotic, poorly vascularized, contain high interstitial fluid pressure, and consequently exhibit low EPR effects and low nanotherapy accumulation 6. In mice, the EPR effect can be dependent on tumor associated MΦ (TAM) accumulation 7, 8, and in patients MΦ imaging positively correlates with initial nanotherapy response 9. Past clinical trials, including one that studied a small cohort of melanoma patients, have found correlation between MΦ infiltration and poor response to immune checkpoint blockade using the programmed death 1 (PD1) targeted monoclonal antibody (mAb) nivolumab 10. Additionally, in a small study of 9 patients with melanoma, kinase inhibitor response correlated more negatively with the drug-induced accumulation of MΦ than any other immune cell-type assessed 11. These instances of clinical correlations and many others suggest that MΦ quantification may be useful in predicting responses to a variety of different drugs 12.

The ability of phagocytes including MΦ to efficiently accumulate nanoparticles has been leveraged for quantitative MΦ-selective imaging in cancer, inflammation, and infectious diseases 13-18. The carboxymethyl dextran coated iron oxide nanoparticle ferumoxytol (Feraheme) is one example of a clinical agent used to image MΦ in patients. Ferumoxytol is approved by the U.S. Food and Drug Administration (FDA) for the treatment of iron deficiency anemia in patients with chronic kidney disease, and is used on an investigational basis to image MΦ by magnetic resonance imaging (MRI) 9, 19-22. Other examples include the crosslinked polyglucose nanoparticle Macrin, which shares similar size (~20 nm) and carboxymethyl dextran components as ferumoxytol, but which can be imaged by ^64^Cu positron emission tomography / X-ray computed tomography (PET/CT), for instance to visualize disseminated metastatic lung cancer 15. Numerous other promising MΦ imaging agents have been described and are extensively reviewed elsewhere 22-26.

Clinical imaging modalities are unfortunately limited in their ability to simultaneously measure large panels of molecular markers. One advantage of imaging general cell-types such as MΦ is that they participate in disease processes through multiple molecular pathways, and thus may apply to diverse applications. It is clear that multiple overlapping molecular pathways may contribute to a given disease or drug-response process, and MΦ quantification may better capture a composite description of these pathways compared to individual measurement of a single molecular feature. The corresponding trade-off is loss in detailed molecular information that can translate into mechanistic understanding. Even “targeted” nanoparticles and imaging agents inevitably accumulate at some level in off-target cell populations due to promiscuous target expression and complex cell-uptake pathways 27. Therefore, a need arises to understand which cellular subsets are actually being imaged by a given probe, and what molecular pathways are operating to influence disease or drug-response processes. It is encouraging that highly multiplexed single-cell analytical technologies, particularly single-cell RNA sequencing (scRNAseq), have emerged as naturally complementary approaches to address these knowledge gaps.

The commercialization of scRNAseq and other single-cell “-omic” methods, combined with large-scale consortium efforts such as the Human Cell Atlas Project to deploy them, has driven growth in the amount of publicly available data in useable formats. The scale of these datasets has increased over time, with ~4,500 cells representing a cutting edge dataset in 2016 28, to 700,000 cells in 2020 29, albeit at diminished sequencing depth per cell in the latter. These datasets can provide broad perspective into the cell subsets that express a receptor of interest, give insight into the molecular programs co-expressed in those cell types, and increasingly support incorporation of post-transcriptional regulation 30, functional genomics 31, spatial localization 32, and drug or probe uptake 33.

With respect to MΦ imaging, scRNAseq has been especially valuable in highlighting distinct MΦ polarization states, patterns of multicellular communication, and pathways of MΦ-influenced drug action within tissues 34-37. MΦ imaging and companion therapeutic applications can be categorized by the class of MΦ receptors or pathways being targeted, and this manuscript is organized accordingly. The review focuses on key MΦ receptor families and pathways of direct material uptake, followed by discussion of MΦ-mediated drug metabolism and indirect effects on drug action via multicellular signaling. Growing abilities to contextualize MΦ imaging with scRNAseq promises to yield mechanistic insights and theranostic design opportunities.

## Mechanisms of material uptake by phagocytes

Biologics, nanoparticles, and protein-bound small molecule drugs are generally internalized into cells *via* endocytosis pathways of phagocytosis (endocytosis of >500 nm diameter particles), macropinocytosis (fluid endocytosis, “cell drinking”), receptor-mediated (clathrin-mediated) endocytosis, and calveolae. Although calveolae have been reported in subsets of myeloid cells such as certain MΦ populations in the lung 38, in general the expression of calveolins (CAV1-3) is much lower in immune cells 37. Conversely, phagocytes are appropriately named for their high capacity for phagocytosis, and also display constitutive and regulated macropinocytosis and receptor-mediated endocytosis. Receptor-mediated endocytosis pathways are especially relevant to the pharmacokinetics (PK) of biologics and nanoparticles, and depend on several families of MΦ-expressed receptors.

### Pathways of receptor-mediated uptake

**Scavenger and lipid receptors.** MΦ remove aged reticulocytes and recover iron, which is essential for heme-containing cytochrome and metalloflavoprotein (e.g. xanthine oxidase) enzymes that metabolize most small molecule drugs 39. The hemoglobin scavenger receptor CD163 (scavenger receptor cysteine-rich type 1 protein, SCARI1) is a MΦ lineage marker found on inflammatory and tissue-resident MΦ 35. Anti-CD163 mAbs have been used to deliver drugs to MΦ both by antibody-drug-conjugate (ADC) and nanoparticle formulations 40, 41. MΦ engulfment of aged red blood cells can divert iron into transferrin for immediate systemic release, or ferritin for storage and slow release, and this decision influences systemic iron levels 42. Iron scarcity increases expression of transferrin receptor, an iron-shuttling protein whose transit across the blood-brain barrier has been extensively studied as a bispecific antibody target to carry cargos for central nervous system applications 43.

**Figure 1:**
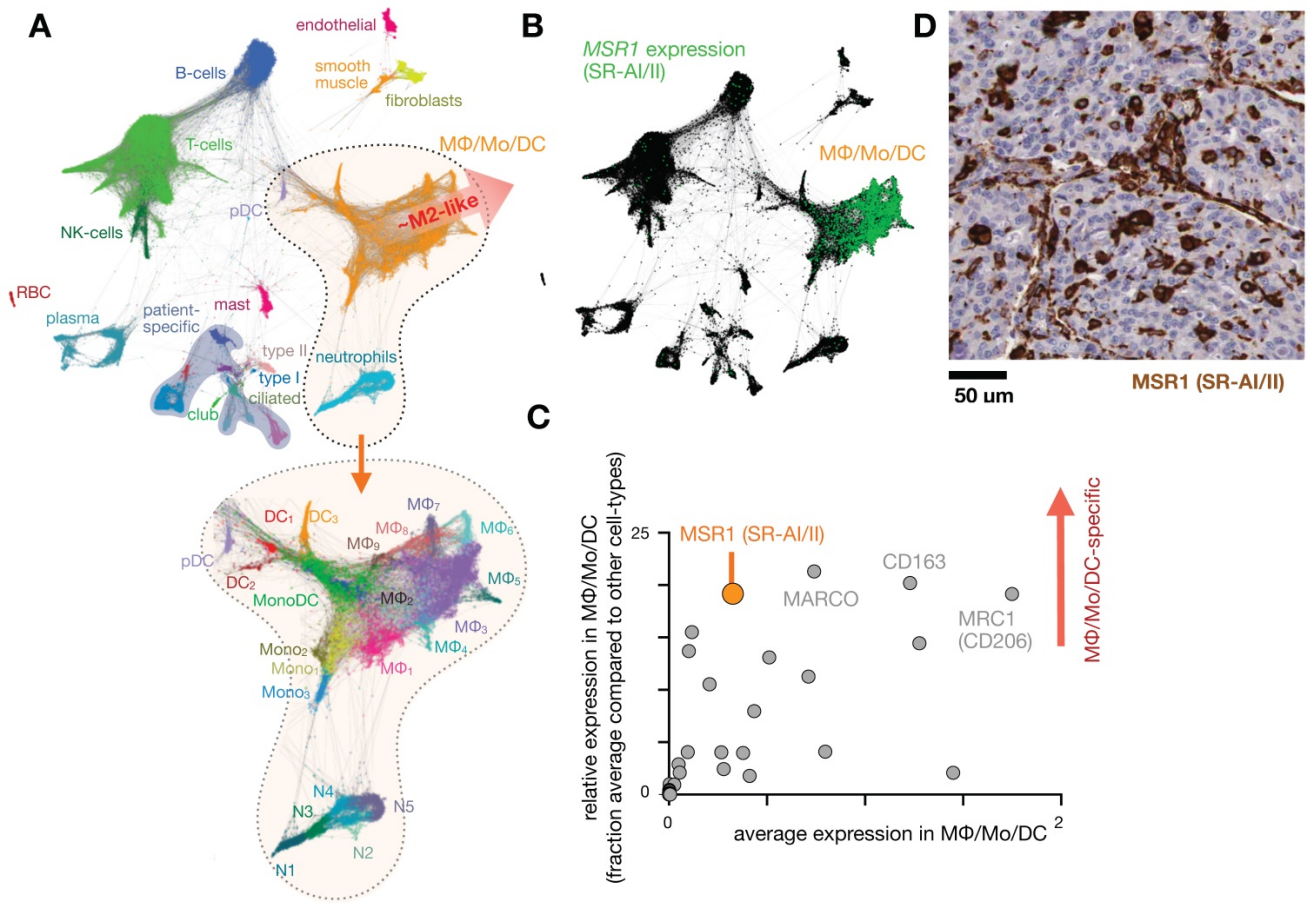
** Expression of scavenger receptors in lung tumors. A)** Single-cell RNAseq was pooled from 7 patients with lung cancer and automatically clustered according to gene expression profile (and thus cell-type) using the SPRING algorithm. Each data point represents a single-cell. At bottom, magnification of the MΦ/monocyte/DC cluster can be further categorized into polarization subtypes. From GSE127465 and SPRING analysis in 37. **B)** Corresponding to *A*, expression of the scavenger receptor type a (SR-AI/II, also known as MΦ scavenger receptor 1, MSR1) is shown in green. **C)** From data in *A*, a panel of 30 scavenger receptors 194 is plotted as a function of average (x-axis) and relatively selective (y-axis) expression in the MΦ/Mo/DC cluster. **D)** Immunohistochemistry of MSR1 in a biopsied lung adenocarcinoma, showing staining consistent with myeloid expression (from the Human Protein Atlas v19.3 195).

**Figure 2:**
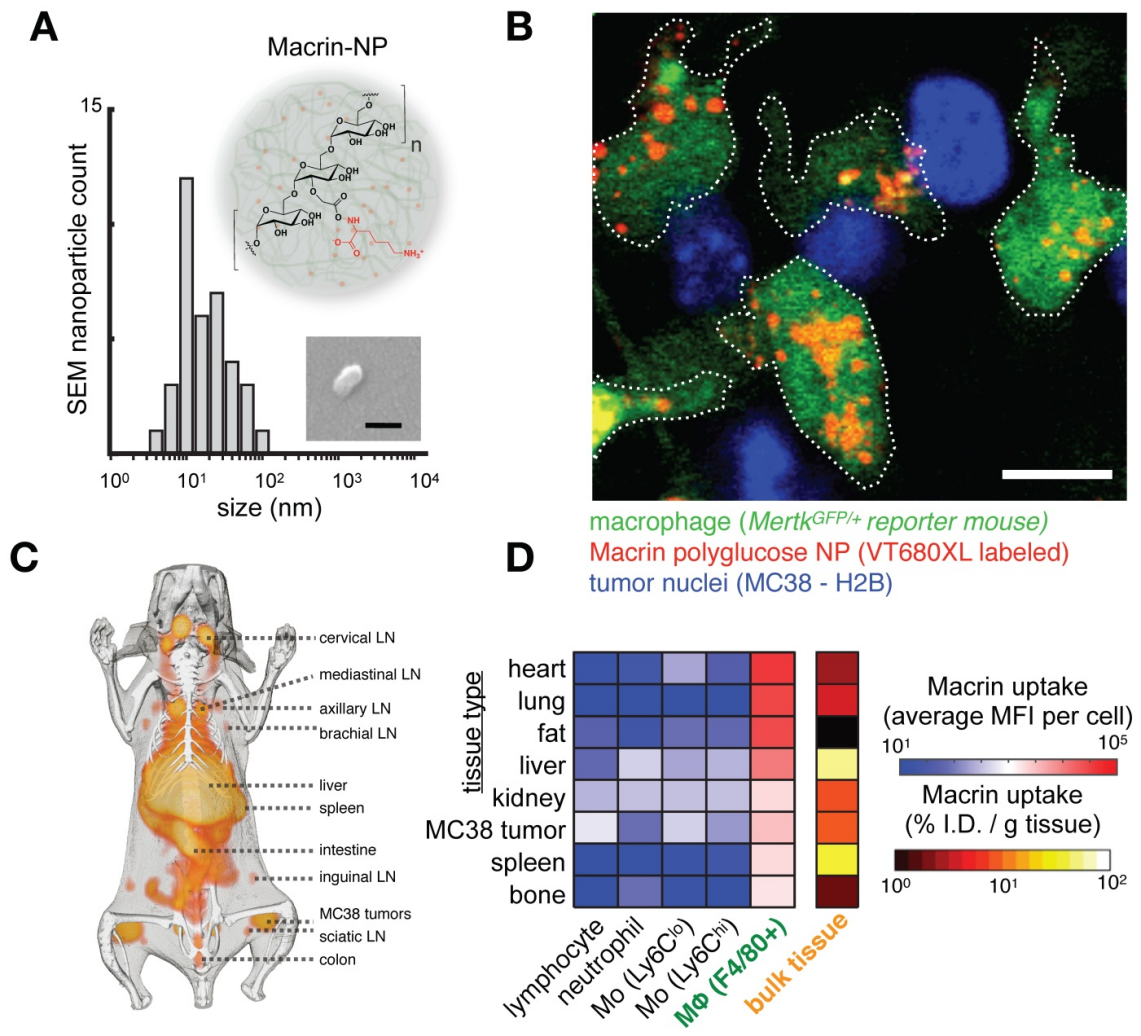
** The dextran-based nanoparticle Macrin accumulates selectively in MΦ in tumor and healthy tissue. A)** Scanning electron microscopy of Macrin, a ~20 nm diameter crosslinked polyglucose nanoparticle that can be labeled with fluorescent probes or the radionuclide ^64^Cu. Scale bar, 20 nm. **B)** Confocal microscopy shows selective Macrin accumulation in tumor associated MΦ, in tumors growing in the *Mertk^GFP/+^* reporter mouse model, which contains GFP+ MΦ. Scale bar, 10 μm. **C)** Positron emission tomography / X-ray computed tomography (PET/CT) of ^64^Cu-labeled Macrin in mice bearing MC38 tumor allografts. **D)** Using the same allograft model, fluorescent Macrin was analyzed for uptake on a per-cell basis by flow cytometry. In all tissues, Macrin accumulation was highest in MΦ, and total organ uptake correlated with MΦ density in tissues rather than uptake on a per-cell basis. All adapted with permission from 15, copyright 2018.

Intolerance to oral ferrous sulfate requires some iron deficient patients to receive parenteral iron dextran or polymerized carbohydrate:iron emulsions. The paramagnetic properties of such agents have allowed researchers to study MΦ distribution, abundance, and function non-invasively using magnetic resonance imaging (MRI), as reviewed previously 19. The receptors responsible for the uptake of iron-containing particulates are many, as MΦ are specialists in recognizing carbohydrate motifs such as chitin, mannose, nucleic acids, and polyglucose 44, 45. For instance, inhibition of macrophage scavenger receptor 1 (MSR1, also known as CD204 and scavenger receptor type AI/II, SR-AI/II; **Fig. 1**) has been shown to block uptake of the dextran-coated nanoparticle ferumoxytol mentioned above, and MSR1 interaction is linked to the anionic carboxymethyl groups of ferumoxytol 46. scRNAseq from lung cancer biopsies suggest MSR1 is expressed across multiple MΦ subsets, but at lower levels in other phagocytes including monocytes, dendritic cells (DCs), and neutrophils (**Fig. 1b-d**). Another example MΦ imaging agent is Macrin, a polyglucose nanoparticle with similarities to ferumoxytol in size, shape, and carboxymethyl dextran composition. Macrin has been used to image tissue MΦ by PET/CT with >90% selectivity via ^64^Cu radiolabeling (**Fig. 2**) 15. Its accumulation in untreated and cytokine-treated MΦ demonstrates its utility for labeling MΦ regardless of the cell state 15.

MΦ activity varies across a spectrum of activation states regulated by signaling and pattern recognition cues 47. A diverse set of polarization states can be identified in TAM by scRNAseq (**Fig. 1a**), yet for simplicity they are typically referred to along an M1/M2 dichotomy. The pro-inflammatory “M1” phenotype, often described as an antigen-presenting subset linked to better prognosis in solid tumor settings, is induced by inflammatory signals such as tumor necrosis factor alpha (TNFα), toll-like receptor (TLR) ligands, and the NFκB (nuclear factor kappa-light-chain-enhancer of activated B cells) pathway. In contrast to pro-inflammatory MΦ, alternatively-activated “M2-like” MΦ arise in wound-healing responses, are frequently found in tumors, and are more generally immunosuppressive given their moderate presentation of antigen and secretion of factors that limit T cell expansion 48, 49.

Transcriptional studies of M2-like MΦ have identified the mannose receptor MRC1 as a useful marker to distinguish this population from M1-like MΦ 50. Chitin itself, an essential structural biopolymer of fungi, helminth and crustaceans, can alternatively activate and polarize MΦ from immature into M2 states^51^. The activity of C-type lectin receptors 52 likely influences the body's response to nanoparticles whose formulations include ligands for these receptors. Given the differences in scavenger receptor expression in different inflammatory contexts, special attention should be paid to ensure the proper MΦ-homing strategy is employed.

MΦ accumulation in the cholesterol-rich regions of atherosclerotic plaques has led to further research into the impact MΦ play in lipid scavenging and metabolism. MSR1 recognition of oxidized low-density lipoprotein (LDL) regulates the activation of antigen presenting cells in the central nervous system and in cardiovascular plaques 53. Unregulated uptake of lipids by atherosclerotic MΦ exacerbates disease and has sparked interest in nanoparticles to saturate MSR1 and its close relative CD36 (also known as scavenger receptor class B member 3, SCARB3) to block foam cell formation 54, 55. These scavenger receptors have also been co-opted into imaging contrast agents for cardiovascular diagnostics 56. In oncology, a myeloid signature containing MSR1 and CD163 was predictive of hepatocellular carcinoma prognosis 57. From the therapeutic angle, synthetic HDL-based nanoparticles have been used to deliver paclitaxel to scavenger receptor class B type 1 (SRB1)-expressing cells 58. The accumulation of fatty acids into lipid droplets is an early indicator of cell stress, and can lead to the sequestration of lipophilic drugs and exacerbation of chemoresistance 59, 60. Lipid uptake therefore offers both an avenue and a roadblock for therapeutic intervention.

The impact of lipid scavenging receptors on local immune responses can differ depending on context. For example, a DC-targeted nanoparticle vaccine was found to induce CD36 and interleukin 10 (IL10), leading to the counter-intuitive suppression of the adaptive immune response 61. Macrophage receptor with collagenous structure (MARCO) is another pattern recognition receptor and lipid scavenger, whose expression has been found by single cell transcriptomics on both alveolar and monocyte-derived MΦ subsets in the lung 62. The observation of MARCO expression on M2 TAM led researchers to develop an agonist MARCO mAb, which increased the efficacy of anti-CTLA4 therapy in an FcγR2b-dependent manner 63. MARCO has been shown to be involved in the uptake of many advanced materials, including silver nanoparticles 64, carbon nanotubes 65, dextran-coated superparamagnetic oxide nanoparticles 66, and polystyrene particles 67. For gene therapy, cellular uptake of lipid nanoparticle delivery systems relies on physical association with apolipoprotein E (ApoE) and lipoprotein receptors. The LDL receptor (LDLR) and LDL receptor-related protein 1 (LRP1) receptors, for instance, substantially contribute to uptake of siRNA-encapsulated lipid nanoparticles 68. During the design of gene therapy systems careful attention also needs to be paid to foreign body responses, as in vivo modification of nanoformulations can affect their distribution, efficacy, and safety.

**Figure 3:**
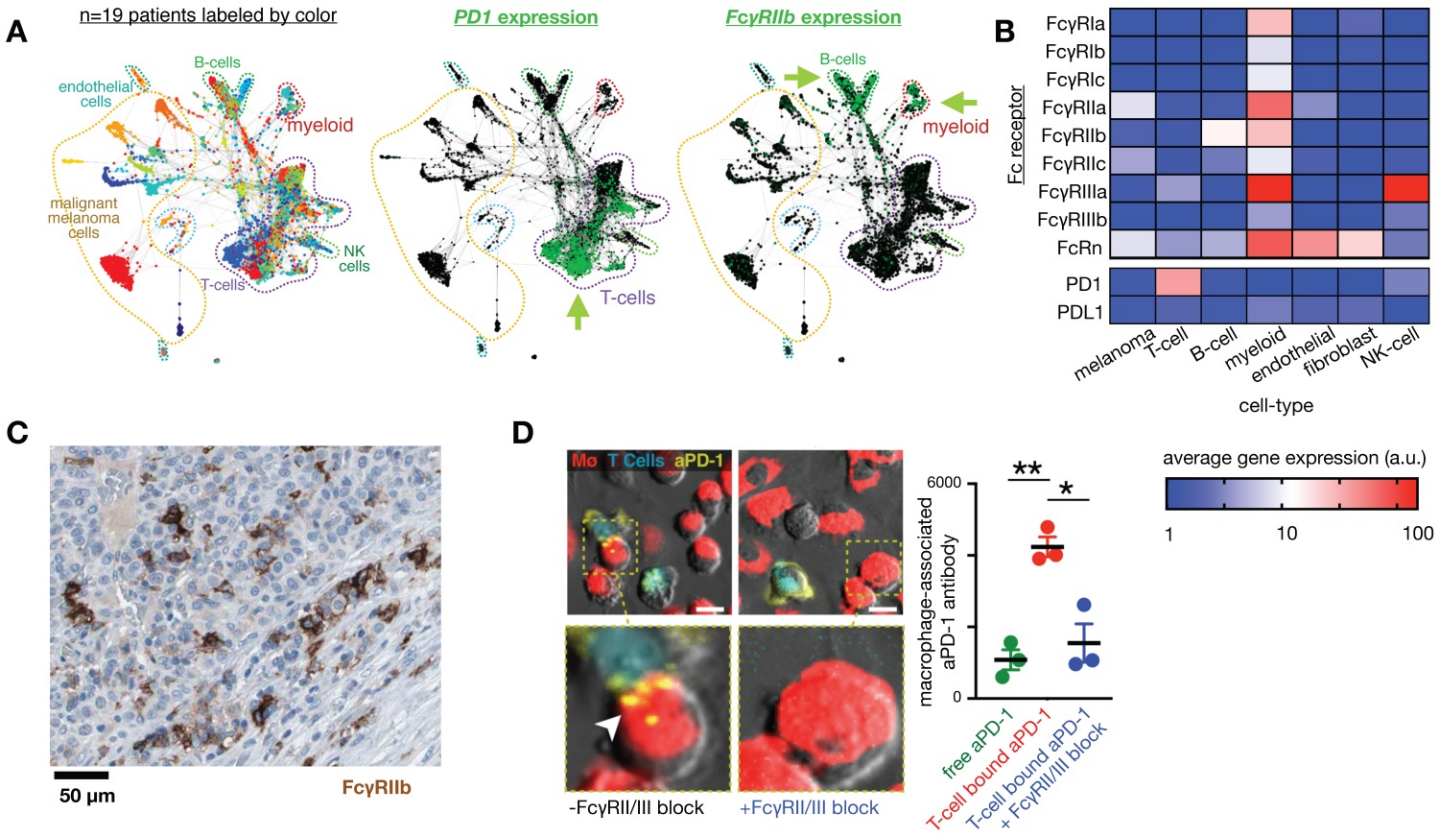
** Fc receptors are highly expressed in myeloid cells and can impact the cellular biodistribution of an anti-PD1 therapeutic antibody. A)** Single-cell RNAseq data from >4,500 cells of melanoma biopsies was clustered according to gene-expression (and thus cell-type), and expression of PD1 and FcγR2b are shown by green color. In this patient cohort, expression of the target of nivolumab, an IgG4 anti-PD1 antibody, is primarily found in T-cells, while its inhibitory Fc receptor FcγR2b is primarily found in B-cells and myeloid cells, including MΦ. Figure adapted with permission from 11 using scRNAseq data GSE72056 28, copyright 2020. **B)** Average expression values corresponding to single-cell data in *A*. **C)** Immunohistochemistry of a melanoma metastasis to the pancreas shows high FcγR2b expression in cells consistent with infiltrating myeloid cells (from the Human Protein Atlas v19.3 195). **D)** 30 minute time-lapse microscopy of co-culture using PD1+ T-cells, MΦ, and anti-PD1 antibody shows transfer of antibody to MΦ from T-cells that were pre-treated with the antibody, in a FcγR2/3 dependent manner. Scale bar 10 μm. Adapted with permission from 80, copyright 2017.

**Fc receptors.** Antibodies consist of fragment antigen-binding (Fab) variable regions at their head and a constant fragment crystallizable (Fc) region at their stem. The Fc region binds complement proteins and cell receptors, including Fc receptors (FcRs) on immune cells (**Fig. 3**). Immune responses to antibody-bound antigen are tailored by the antibody class and its avidity for each of the 11 total FcRs in humans. Antibody-producing B cells undergo class switching to control the immunoglobulin (Ig) isotype (IgA1-2, IgD, IgE, IgG1-4, and IgM) and its corresponding effector function. Each Ig isotype differs in its avidity for each FcR, introducing a complexity suitable for handling diverse pathogenic challenges. For therapeutics, subclasses of the IgG type are used with few exceptions. IgG antibodies communicate neutralizing instructions through the Fc-gamma receptor (FcγR) family, the members of which exhibit distinct avidity for IgG subclasses and distinct downstream activities. IgG antibodies are long-circulating, have a well-understood manufacturability, and contain a diverse enough repertoire of effector functions such that antibody-dependent cell-mediated cytotoxicity (ADCC), phagocytosis, complement recruitment, signal blockade, and even receptor agonism can be achieved with this isotype alone 69.

**FcRn.** Monoclonal antibodies (mAbs) are constantly internalized and recycled by cells due to a mechanism wherein acidic intracellular conditions thermodynamically favor neonatal Fc receptor (FcRn) binding, leading to exocytosis, diminished FcRn affinity at neutral pH, and recirculation. This process contributes to the long circulation half-lives (>3 weeks) of most mAb therapies, and has inspired the common practice of Fc-fusion for extending circulation 70. Albumin also binds FcRn, and consequently albumin binding is a successful strategy to extend PK of therapeutics 71 and imaging agents 72. FcRn expression has been observed on the endothelial cells that line blood vessels as well as the serum-sampling perivascular MΦ that help shape vessel structures 73. FcRn expression on gut epithelial cells has been used to orally deliver IgG-Fc coated nanoparticles for systemic distribution 74. Recently, scRNAseq has made possible detailed queries into FcRn involvement in local, diseased tissues (**Fig. 3**). Efgartigimod, one of several FcRn blocking agents at various stages of development, has shown efficacy in a Phase III trial to treat the autoantibody-driven immune disorder myesthenia gravis (NCT03770403) 75. New insights into FcRn involvement in local distribution of therapeutic mAb requires development of mAb labeling techniques that don't interfere with the FcRn binding domain of the Fc region, although labeled albumin can be a useful surrogate 76.

**Figure 4:**
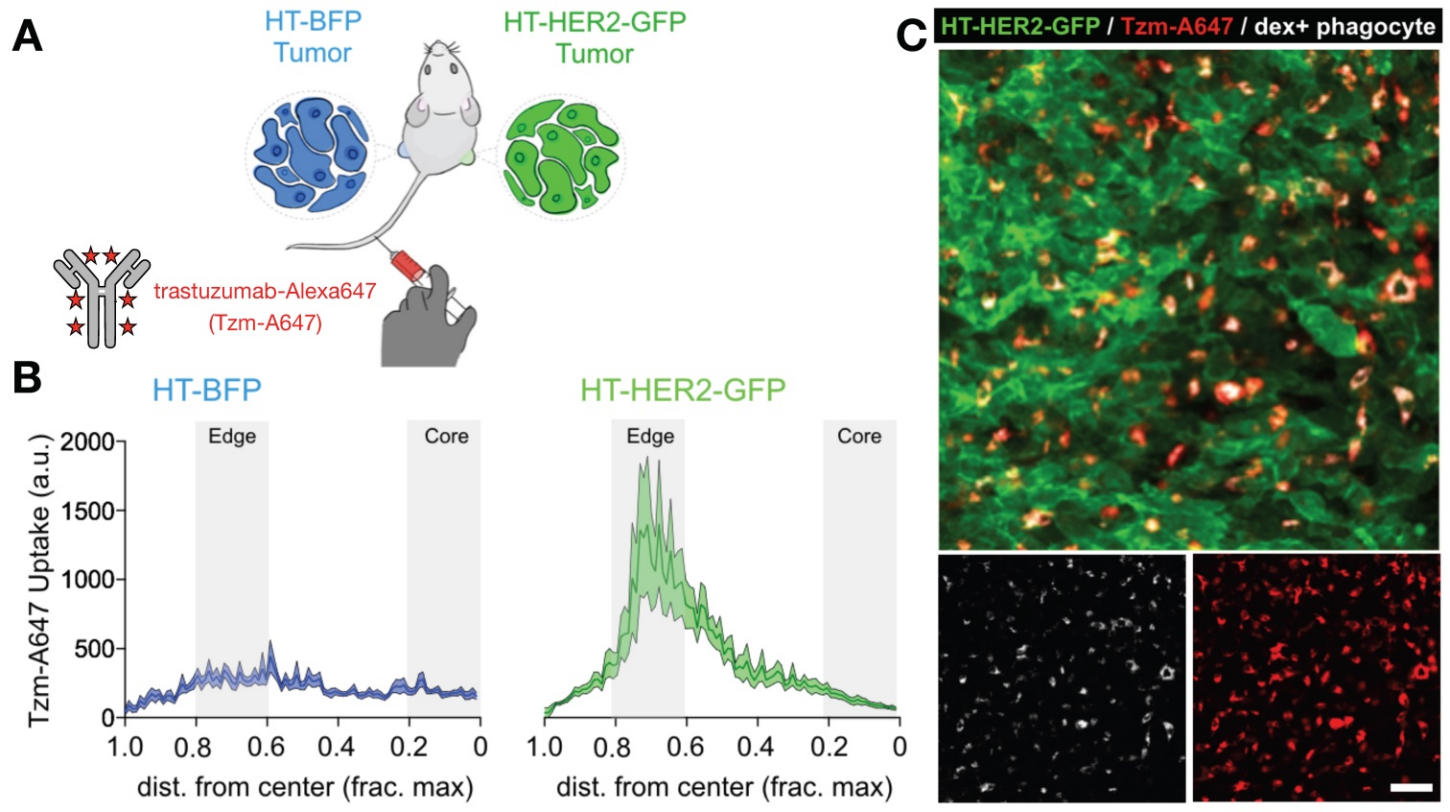
** Matched HER2+ and HER2- xenografts show target-dependent and -independent antibody uptake into tumor associated phagocytes. A)** Contralateral xenografts were grown either with transgenic expression of a HER2-GFP fusion protein (HT-HER2-GFP), or with blue fluorescent protein lacking HER2 as a control (HT-BFP). Fluorescently labeled anti-HER2 antibody trastuzumab (Tzm-AlexaFluor647) was injected intravenously and tissue was collected the following day. **B-C)** Quantification (**B**) and corresponding representative confocal microscopy (**C**) revealed that trastuzumab accumulated in both tumor-types, but at higher levels in the HER2+ tumors. However, by 24 h most antibody was in phagocytes including MΦ, rather than on HER2+ tumor cells. Scale bar, 50 μm. Adapted with permission from 81, copyright 2020.

**FcγR.** mAb effector activity is carried out by proteins and FcγR-expressing cells that recognize the Fc region of an antigen-bound mAb (**Fig. 3a-c**). The high-affinity FcR, FcγR1 (CD64), tightly binds monovalent IgG, is expressed highest on MΦ, and has been described as a reliable MΦ-specific marker in both mouse and human transcriptomic studies 77. In contrast, FcγR2a and FcγR3 have higher binding affinities for IgG that have clustered into immune complexes^78^. FcγR3a (CD16) is commonly included in flow cytometry staining panels for NK cells, however, this would not be a reliable target for NK imaging agents as scRNAseq suggests multiple myeloid populations to also be high expressers (**Fig. 2**). These activating Fc receptors are the promoters of antigen-destruction pathways because they convert adaptive immune signals (e.g. IgG1s) to recruit innate immune cell killing. Not all mAbs form effective low-affinity FcγR-activating immune complexes, and some (e.g. IgG4, deglycosylated mAbs, Fc-null or Fc-effectorless mAbs) act instead to bind and block extracellular signal transmission 79. FcγR2b is unique in that it has an ITIM (immunoreceptor tyrosine-based inhibition motif) domain in place of the activating ITAM (immunoreceptor tyrosine-based activation motif) found on the intracellular region of most FcγRs. Expressed in B cells and myeloid cells (**Fig. 2**), FcγR2b is an inhibitory receptor capable of binding and mechanically engaging mAb-bound receptors, without activating a destructive immune response against the antigen in question. In mice, tumor associated MΦ (TAM) express especially high FcγR2b 63, and imaging studies have found that TAM can strip an anti-PD1 mAb from the surface of PD1+ T-cells in a FcγR2/3-dependent process (**Fig. 3d**) 80. In vivo tumor imaging revealed anti-PD1 mAb initially on PD1+ T-cells, but showed a gradual shift to accumulation within TAM. FcγR blockade enhanced anti-PD1 response in the corresponding mouse allograft model 80. Similar imaging studies with fluorescently-labeled anti-HER2 mAb trastuzumab have found initial accumulation on HER2+ cells, but by 2 days after injection, the localization was primarily in TAM rather than tumor cells (**Fig. 4**) 81. The mAb isotypes used in these studies were distinct, as were mouse strains, and FcR-dependencies therefore are likely to be different. Future work needs to map how isotype and Fc-engineering influence TAM uptake dynamics and productive effector functions such as ADCC and antibody-dependent cell phagocytosis. Molecular imaging to selectively study FcγR2b-expressing cells would be useful for engineering better non-depleting agonist mAbs.

**MΦ polarization-influenced FcR activity.** Pro-inflammatory signals can upregulate MΦ FcRn expression and thus accelerate the rate of mAb recycling in M1-like MΦ 82. Interferon-gamma (IFNγ), a key signal in the induction of pro-inflammatory phenotypes, influences the fate of internalized material. M1-MΦ, partly due to their higher IFNγ-induced nitric oxide synthase 2 (NOS2) expression, generally are slower to degrade phagocytosed material 83. Endocytosis can occur through FcγR binding, and FcRn, in this instance, salvages FcγR-bound antigens to ensure processing for MHC loading and presentation to the adaptive immune system 84. MHC expression is also IFNγ-inducible, and the loss of antigen presentation machinery in TAM has led to many “re-polarization” efforts for solid tumor therapy 85, 86. In contrast to pro-inflammatory MΦ, IL-4 induces precursor monocytes to mature M2-like MΦ and counteracts IFNγ by lowering FcγR1 expression 87. The kinetics of phagosomal acidification and proteolysis are faster in M2-MΦ, and pH-sensitive materials have been used as imaging agents for this subset 88, 89. MΦ polarization also influences expression of other degradative components including proteases 90.

**Figure 5:**
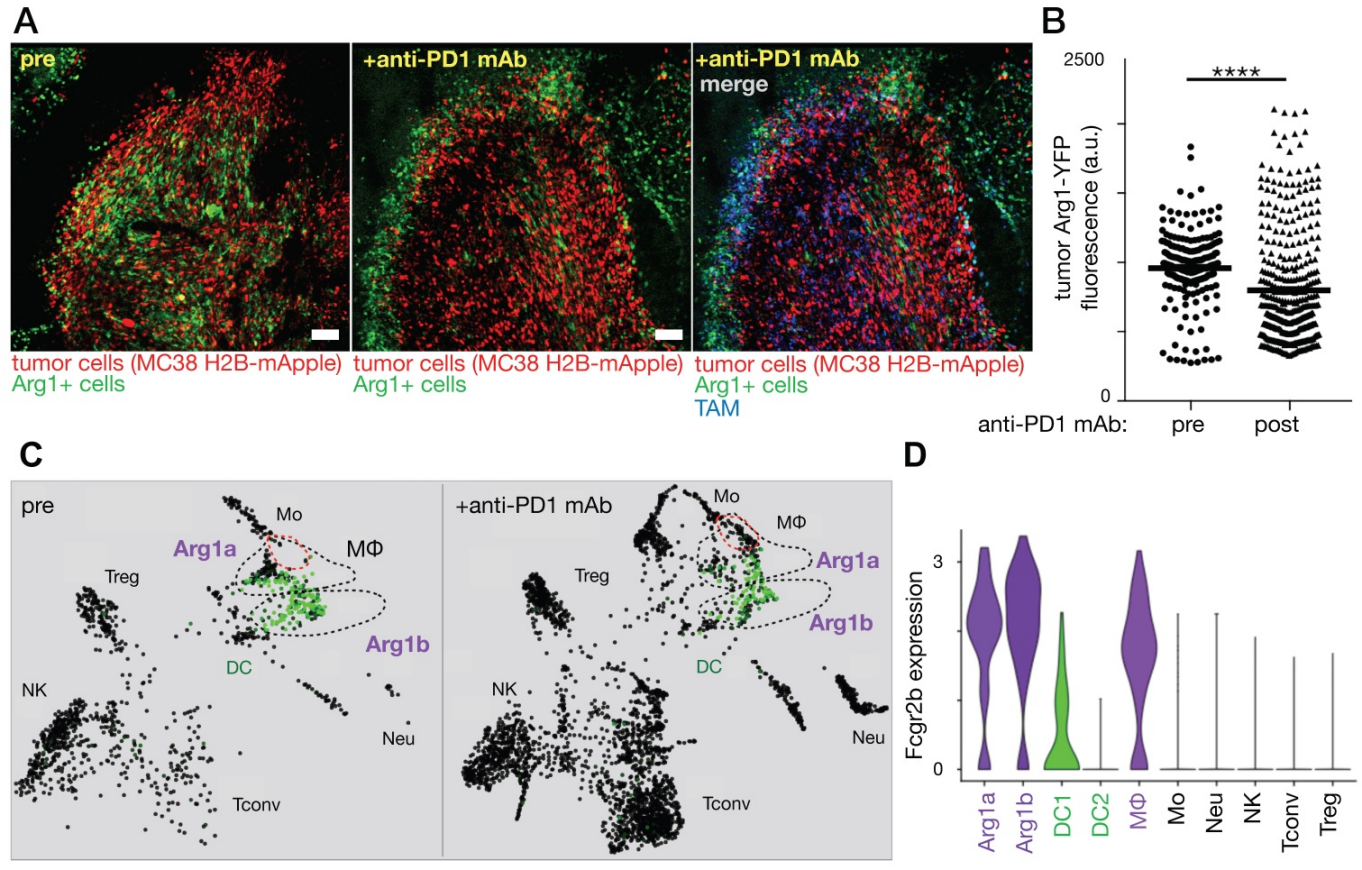
** Immune checkpoint blockade shifts TAM polarization and spatial infiltration. A-B)** Intravital microscopy was used to assess allografts of the MC38 cancer cell line growing in a genetic reporter mouse model where yellow fluorescent protein was driven by arginase 1 (Arg1) promoter activity. Images before and 3 days after anti-PD1 antibody treatment show decreased Arg1 expression. Scale bar, 100 μm. **C)** The SPRING algorithm was used to cluster scRNAseq using the same allograft model into immune cell-types, revealing Arg1 expression (green) primarily in TAM subsets labeled Arg1a and Arg1b. **D)** Violin plots from scRNAseq data show both Arg1a and Arg1b subsets express high levels of Fcgr2b compared to other immune subsets. Adapted with permission from 94, copyright 2018.

MΦ activation states have been transcriptionally defined, and IL12b and Arg1 have been used to define M1-like and M2-like MΦ, respectively, in mice 91, 92. In vivo confocal (intravital) microscopy of tumors embedded intradermally in IL12-eYFP reporter mice revealed the additive effect M1-inducing therapies can have when delivered in combination with immune checkpoint blockade using anti-PD-1 mAb 93. Conversely, effective anti-PD-1 response was observed by intravital microscopy to include replacement of Arg1-expressing TAM with highly-motile pro-inflammatory monocytes and M1 precursors (**Fig. 5**) 94. Despite observed differences in polarization, both Arg1-expressing subsets were found to exhibit high FcγR2b expression (**Fig 5**).

**Complement Receptors.** Complement factors are another means by which a particle or cell can be marked for phagocytosis. Upon injection, nanoparticles form a protein corona as they interact with serum proteins that adsorb on their surface 95. As the protein corona evolves, particle opsonization with complement proteins can stimulate phagocytosis. C1q is a component of the classical complement pathway and is one of the strongest differentially-expressed genes that distinguishes pro-inflammatory MΦ from their tolerogenic counterparts in the liver 35. C1q has a broad range of binding partners including IgG, IgM, and phosphatidylserine (PS), and its role in clearing apoptotic cells is critical for prevention of autoimmunity 96. Complement recruitment is a major effector function by which the anti-CD20 mAb rituximab works in the treatment of B-cell malignancies and autoimmune disorders, but it also represents a source of heterogeneity in the effectiveness and occurrence of adverse events among patients 97, 98. In part to address this issue, an anti-CD20 mAb obinutuzumab was Fc-engineered to bypass complement-directed cytotoxicity and was shown by intravital microscopy to enhance the antibody-directed phagocytosis of B lymphoma cells by Kupffer cells, a liver-resident macrophage population 99. Alternative opsonins to C1q, such as mannose-binding lectin, also exist. Myeloid cells serve as innate immune effectors for the complement pathways and express receptors that can initiate chemotaxis, phagocytosis, and degranulation activity. Platelet activation through complement can cause fatal intravascular coagulation and has been shown to have a major influence on nanomaterial hypersensitivity 100, 101. Cationic surfaces exacerbate this hemolytic effect and enhance corona formation, thus a slight negative charge usually imparts stronger tumor-homing capabilities 6. Corona and complement formation also offer an indirect avenue through which material uptake by MΦ can occur.

**Figure 6:**
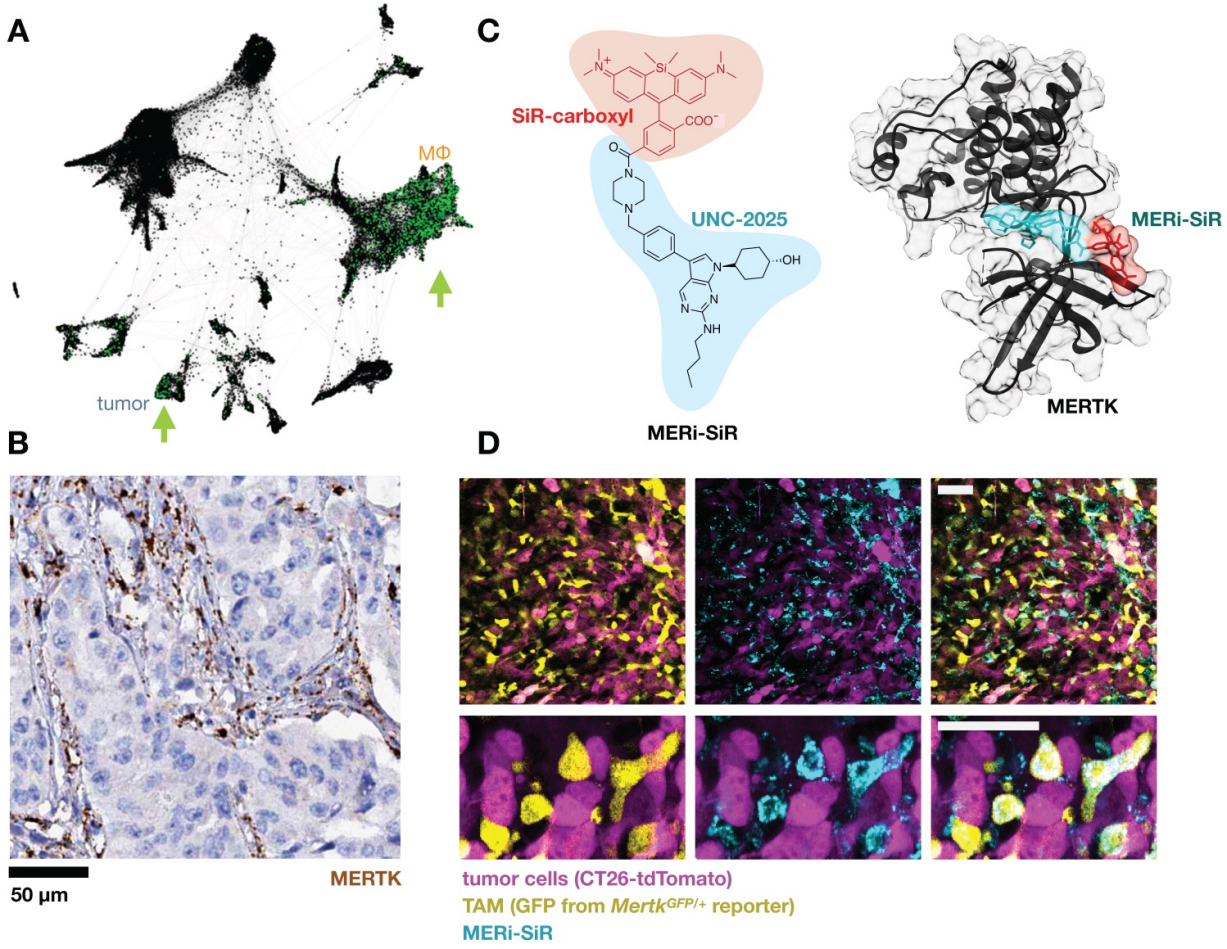
** Targeting the efferocytosis receptor MERTK to image MΦ. A)** As in Fig. 1, scRNAseq was pooled from 7 patients with lung cancer and *MERTK* expression is shown in green. Some patients show *MERTK*+ cancer cells, and *MERTK* is expressed across multiple MΦ subsets. From GSE127465 and SPRING analysis in 37. **B)** Example immunohistochemistry from a lung adenocarcinoma biopsy shows MERTK staining consistent with expression in stromal / myeloid cells rather than malignant cells (from the Human Protein Atlas v19.3 195). **C)** The MERTK kinase inhibitor UNC-2025 was modified with the near-infrared silicon rhodamine COOH (SiR) to yield the fluorescent probe MERi-SiR, shown in a docking simulation bound to MERTK. **D)** Confocal microscopy of CT26 allograft tumors in the *Mertk^GFP/+^* knock-in reporter mouse shows co-localization in GFP expression (which reports on *Mertk* expression) with uptake of MERi-SiR. Scale bar, 50 μm. *C-D* adapted with permission from 107, copyright 2017.

**Efferocytosis receptors.** When a red blood cell ages, it expresses many of the ligands for phagocyte scavenger receptors discussed in previous sections. Additionally, loss of the “don't eat me” signal CD47 overcomes any remaining SIRP1α-mediated inhibition and permits the engulfment of the aged cell and recycling of its components. This pathway is co-opted by malignant cells as a means of immune evasion and has inspired CD47-blocking mAb clinical stage programs from several companies (NCT02953509; NCT03512340; NCT02641002; NCT03013218; NCT02663518; NCT02890368; NCT03530683). CD47 and other marker-of-self signals have also been used to shield nanoparticles to extend circulation 102. When not blocked by don't-eat-me signals, phagocytes respond to PS on the outer bilayer of aged and apoptotic cells by initiating efferocytosis. PS is recognized by a range of receptors and its effects on the phagocyte have been observed to be broadly immunosuppressive 103. TIM1, TIM3 and TIM4 are known PS receptors whose blockade in mice caused autoantibody formation 104. At least 10 other receptors have been described, many highly expressed on MΦ, including the CD300 family and STAB1/2 that directly bind PS. Although not through direct binding, the TYRO3/AXL/MERTK family efferocytosis receptors can be engaged indirectly when bridged by the GAS6 and PROS1 serum proteins, and mAb-based tools have been developed for in vivo imaging of AXL 105. Genetic defects in Mertk can lead to defunct debris clearance and formation of necrotic plaques in mice 106. MERTK has an immunosuppressive effect when engaged on MΦ, and several inhibitors in development have reached clinical trials (NCT01482195; NCT03176277; NCT03176277). One such clinical candidate, UNC-2025, was tagged with a silicon rhodamine and used as an imaging agent to study MERTK receptor distribution in mouse tumor allografts and metastases, revealing high TAM accumulation (**Fig. 6**) 107.

### Macropinocytosis

Macropinocytosis is an endocytic process that allows cells to non-selectively internalize extracellular fluid, solutes, and antigens. It is an evolutionarily conserved form of endocytosis that can be carried out by nearly all types of cells inside the body, including innate immune cells, fibroblasts, epithelial cells, and neurons 108. Macropinocytosis can influence the PK of therapeutic agents, as drugs dissolved in the extracellular space can be non-selectively taken up by cells through this process 109. Macropinocytosis is primarily driven by actin cytoskeleton rearrangement near the plasma membrane 110, which results in the folding back of the membrane to create endocytic vesicles 0.2-5 μm in size. These large endocytic vesicles are called macropinosomes, and they allow the cells to efficiently take up large quantities of extracellular materials, including drugs 111.

Signaling molecules involved in actin remodeling, such as Ras, PI3K, Rac1, Cdc42, and Pak1 influence macropinocytosis 111. Moreover, sorting for the nexin (SNX) family of membrane proteins and C-terminal binding protein (CtBP), which controls membrane trafficking, have been implicated in macropinosome formation 111, 112. Proteins involved in endosomal regulation, such as Rab5 and Rab34, have been shown to associate with macropinosomes 111. Regulators of cellular metabolism, such as AMPK and mTOR, can also influence macropinocytic activity, since cells can use macropinocytosis to take up nutrients and abundant proteins such as albumin in the extracellular space. Functional genetic RNA interference screens have been used to systematically identify regulators of macropinocytosis in RAS-driven cancer cells and MΦ, providing broad perspective into underlying regulatory processes 113. For instance, macropinocytosis contributes to the uptake of low density lipoprotein (LDL) as MΦ transform into foam cells during atherosclerosis 114, and a functional genetic screen showed pathways involved in LDL uptake and foam-cell generation, including CSF1R, CXCR4, and involvement of APOC1 and FABP4 115. While macropinocytosis transcriptional signatures have been linked to drug outcomes, these bulk analyses are unable to elucidate the specific contribution of MΦ to this pharmacology 116.

Macropinocytosis can be stimulated by growth factors in many cell types 110, yet can be constitutive in RAS-mutant cancer cells 117 and some subsets of MΦ 118 and DCs 110. Macropinocytosis mediates immune cell functions such as antigen scavenging and presentation by MΦ and DCs 118, chemotactic migration of neutrophils 119, and proliferation of T cells 120. Macropinocytosis in particular has been shown to mediate uptake of nanomaterials, including lipid nanoparticles (LNPs) 121 and silica-based nanoparticles 122. For instance, it controls the uptake of RNA vaccine by immature DCs in lymph nodes 123. The uptake of viral vaccine vectors by antigen presenting cells, a crucial step in vaccine-induced immunity, is also mediated by macropinocytosis 124. One recent study demonstrated that macropinocytosis drives the uptake of nanoparticulate albumin-bound paclitaxel (nab-paclitaxel, marketed as Abraxane and FDA-approved for treatment of pancreatic adenocarcinoma and other cancers) by TAM, leading to the activation of pro-inflammatory phenotypes in these cells 125. Furthermore, M2-MΦ have been reported to exhibit enhanced macropinocytosis activity compared to those with a pro-inflammatory M1 phenotype 126. Thus, the polarization state of MΦ can impact their macropinocytic activity, which may have ramifications in designing therapeutics to target select MΦ subsets.

### Small molecule transporters

Membrane transporters fall into classification as ATP-binding cassette transporters (ABC transporters), solute carrier family (SLC) transporters, and the superfamily of P-type ATPases. The latter family consists of flippases responsible for maintaining asymmetric phospholipid cell membranes, and importantly ion pumps including ATPase copper transporting alpha and beta (ATP7A and ATP7B), which promote efflux of, and resistance to, the chemotherapeutic cisplatin 127. However, these two genes are not typically expressed in leukocytes at high levels. ABC transporters are implicated in the PK of many drugs, and include family members MDR1 (P-glycoprotein) and MRP1 that can be found expressed in lymphocytes and drug-resistant cancer cells. Although phagocytes express relatively low levels of MDR1 and MRP1, MΦ express ATP-binding cassette transporter ABCA1 (cholesterol efflux regulatory protein, CERP) and ABCG1, which both govern homeostasis of cholesterol and phospholipids. Loss of ABCA1 function leads to the autosomal recessive Tangier disease characterized by abnormally low circulating levels of high density lipoprotein (HDL). ABCA1 has been reported to affect the local, cellular-level distribution of Amphotericin B, a polyene anti-fungal that binds preferentially to membrane sterols, particularly the fungal ergosterol but also cholesterol. ABCA1-expressing cells transport cholesterol to the cell surface where interaction is observed with Amphotericin B, leading to decreased cellular cytotoxicity, which is hypothesized to mitigate its toxic side effects 128.

Both neutrophils and MΦ express ABCG1, and in two independent studies 129, 130 SNPs of the ABCG1 gene were found to associate with toxicity of the chemotherapeutic irinotecan in patients with metastatic colorectal cancer. One study hypothesized this was not due to direct action on irinotecan, but rather to inflammation associated with defective ABCG1 activity, and unbalanced cholesterol homeostasis, as has been observed in mice and patients 130. In the other study, the ABCG1-associated toxicity was severe neutropenia 129. ABCG1 is a confirmed transporter of the chemotherapies mitoxantrone and doxorubicin 131, and the pentacyclic ring structure of irinotecan bears similarity to the ring structures of other ABCG1 substrates.

In comparison to active transport by ATP hydrolysis, SLC transporters use secondary active transport and exploit the membrane potential of ions such as Na^+^ or H^+^. These transporters move drugs across the plasma and organelle membranes. Organic anion transporting polypeptides (OATPs) including OATP1B1 and OATP1B3 are prominent in hepatic and intestinal transport. MΦ express high levels of OATP2B1 (known as SLCO2B1), which is also known to be expressed in other tissues including the brush-border membrane of the small intestine and hepatocyte basolateral membranes 132. OATP2B1 mediates MΦ uptake of the uremic toxin indoxyl sulfate, which is a metabolite of dietary tryptophan and elicits pro-inflammatory signaling and atherosclerosis lesion development in mice 133. Hydroxymethylglutaryl-coenzyme A (HMG-CoA) reductase inhibitors including fluvastatin, rosuvastatin, and the antihistamine fexofenadine are OATP2B1 substrates according to recent reports that examined OATP2B1 knockout mouse models 134.

Expression of SLC15A4, also known as peptide/histidine transporter 1 (PHT1), in myeloid cells is associated with transport of histidine, carnosine, and the antiviral prodrug valacyclovir 135. Other SLC15 family members have also been examined as carriers of prodrugs including valacyclovir 136. In myeloid cells SLC15A4 has been implicated in endolysosomal toll-like receptor (TLR) signaling, autoimmune disease, and downstream activation of the interferon regulatory factor 5 (IRF5) transcription factor which controls MΦ activation 137.

Multi-gene programs of MΦ-expressed SLCs can be dynamically regulated during the phagocytic clearance of apoptotic cells and cell debris (efferocytosis), with prominent contribution noted from SLC16A1 (also known as monocarboxylate transporter 1, MCT1), which can export lactate from MΦ undergoing aerobic glycolysis and contribute to an anti-inflammatory extracellular environment 138. Proliferating T-cells also use aerobic glycolysis, rely on MCT1 for lactate efflux, and MCT1 inhibition can thus be immunosuppressive 139. MCT1 has also been identified in a genome-wide functional screen to mediate uptake of the potential anticancer agent 3-bromopyruvate into cancer cells, where it can disrupt glycolysis 140, and MCT1 has been associated with the transport of drugs including bumetanide, valproic acid, nateglinide, salicylate, simvastatin and atorvastatin 141.

**Figure 7:**
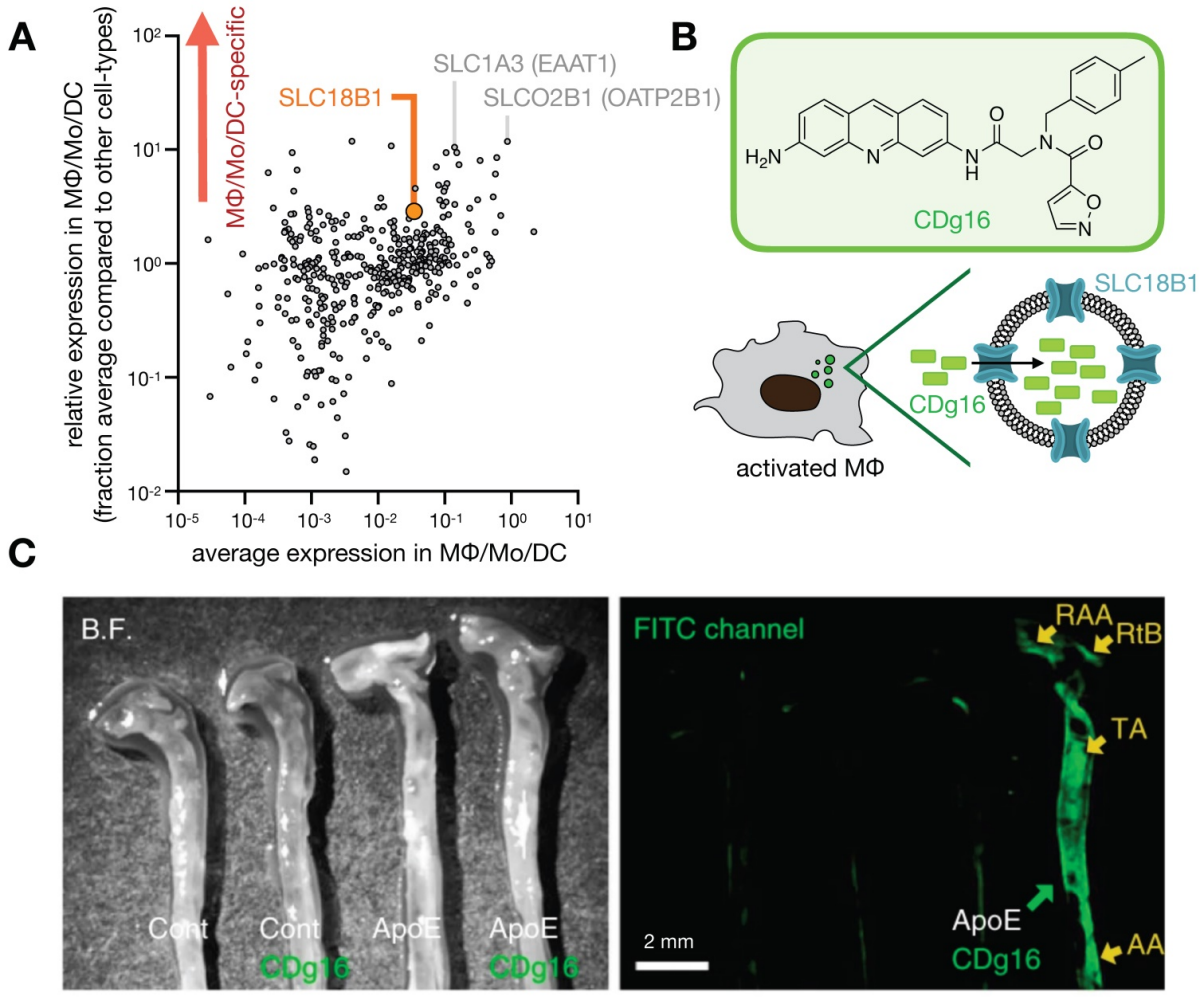
** Probing solute carrier family proteins. A)** As in Fig. 1, scRNAseq was pooled from 7 patients with lung cancer, and expression of 406 solute carrier family (SLC) genes were plotted as a function of average expression (x-axis) and selectivity of expression within the lung (y-axis), among cells in the MΦ/Mo/DC cluster. From GSE127465 and SPRING analysis in 37. **B)** The fluorescent probe CDg16 was identified in a high-throughput screen as selectively accumulating in activated MΦ, and CRISPR screening revealed SLC18B1-mediated uptake 142. **C)** CDg16 was used to image MΦ-rich plaques in the aortas of atherosclerotic ApoE-knockout mice. Excised vessels are annotated with the root of the aorta arch (RAA), the right brachiocephalic artery (RtB), the thoracic aorta (TA), and the abdominal aorta (AA). *C,* adapted with permission from 142, copyright 2019.

Phagocyte-selective expression of SLC transporters has been exploited to develop imaging probes (**Fig. 7**), as recently demonstrated with the fluorophore CDg16 found to accumulate in activated MΦ 142. In this study, fluorescent compounds were first screened for selective accumulation in stimulated MΦ, and functional CRISPR screening revealed uptake was dependent upon SLC18B1. The probe was effective in labeling MΦ within atherosclerotic plaques in ApoE^-/-^ mouse aortas 142.

## Phagocytes and drug metabolism

**Figure 8:**
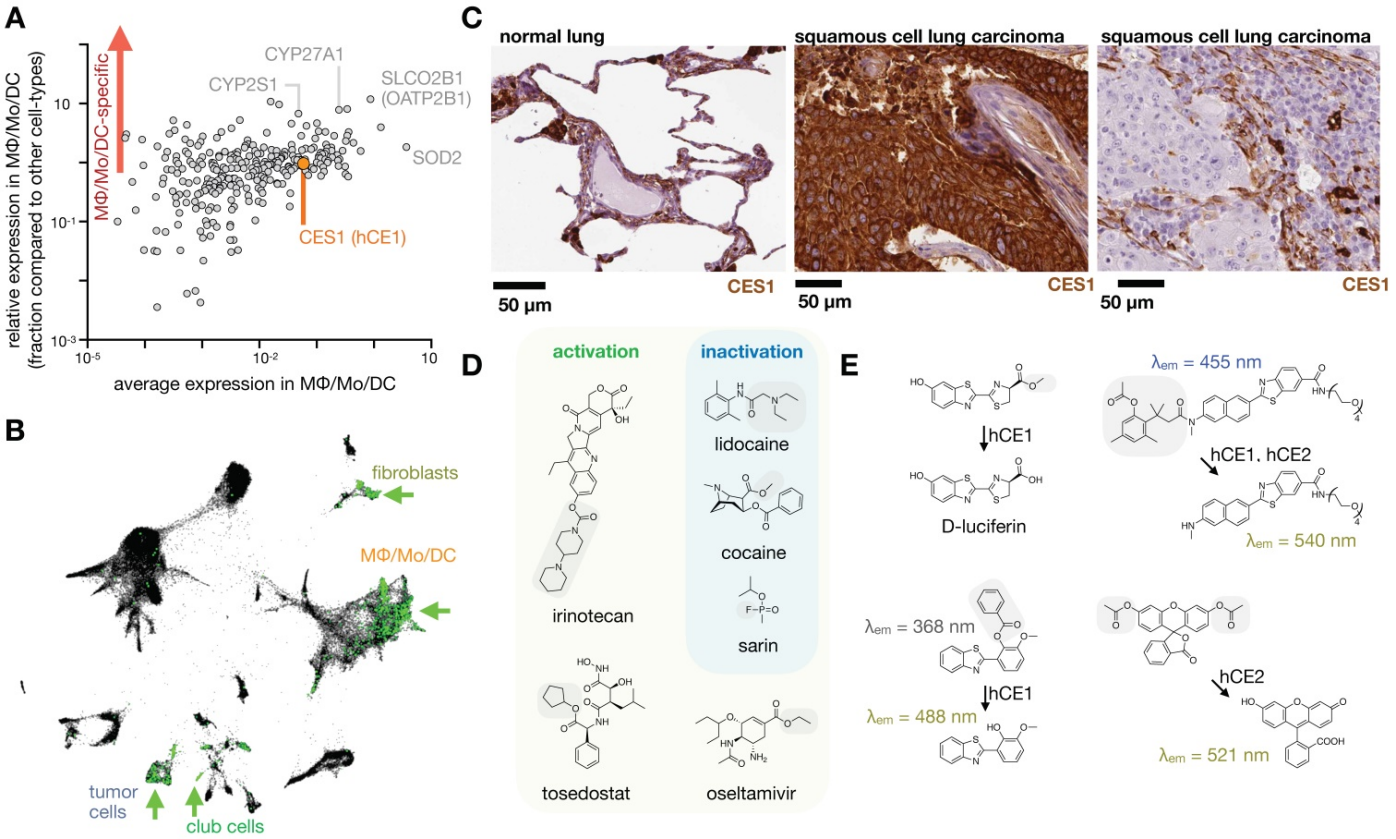
** MΦ expression and imaging of ADME-relevant enzymes and transporters. A)** As in Fig. 1, single-cell RNAseq was pooled from 7 patients with lung cancer, and expression of 288 high-priority genes related to drug PK (www.PharmaADME.org) were plotted as a function of average expression (x-axis) and selectivity of expression within the lung (y-axis), among cells in the MΦ/Mo/DC cluster. From GSE127465 and SPRING analysis in 37. **B)** Corresponding to *A* and Fig. 1, data were clustered according to cell-type, and expression of CES1 (hCE1) is shown in green. Arrows highlight high-expressing populations. **C)** Immunohistochemistry of CES1 in biopsied healthy and malignant lung tissue, showing representative examples of staining consistent with high expression in alveolar MΦ (left), high tumor-cell expression (middle), and high phagocyte or stromal expression (right). From the Human Protein Atlas v19.3 195. **D)** Example CES1-activatable prodrugs, and CES1-neutralized substances, which may be metabolized by phagocytes in the lung. Gray boxes mark site of carboxylesterase cleavage. **E)** Probes have been developed to report carboxylesterase activity, with relative CES1 versus CES2 specificity noted. CES-mediated cleavage yields a functional bioluminescence substrate (top left 196), creates a red-shift in fluorescence following single-photon (bottom left 197) or two-photon fluorescence excitation (top right 198), and turns on fluorescence, as with fluorescein diacetate which is used with purified enzyme 199 or as a readout of cell viability (bottom right).

In addition to mere transport, MΦ express enzymes that can participate in chemical drug transformation (**Fig. 8a-c**), and local MΦ accumulation at sites of disease may allow prediction of corresponding local drug activity.

**Proteases.** Phagocyte-expressed reactive enzymes, including proteases, have been exploited for activation of nanoparticles, antibodies (“pro-bodies”), antibody drug conjugates, and small molecule prodrugs. scRNAseq data reveal distinct patterns of relevant protease expression across cell-types in the tumor microenvironment. In scRNAseq from a cohort of lung adenocarcinoma biopsies, neutrophils and TAMs are the primary expressers of cathepsin B (CTSB) 37. Both neutrophils 143 and MΦ produce matrix metalloproteinase 9 (MMP9) 37, 144. In contrast, other relevant proteases including MMP2 can be more highly expressed in cancer-associated fibroblasts than leukocytes 37. MMP14 (also known as membrane type-I matrix metalloproteinase, MT1-MMP) can be expressed across a mix of fibroblasts, MΦ, and tumor cells 37. Among MΦ, enrichment in tissue-resorbing cathepsins and proteolytic enzymes have been observed by scRNAseq in specialized M2-like wound-healing subsets 90. These proteases and others participate in various disease processes, particularly with respect to remodeling of the extracellular matrix, and are drug targets themselves.

Translational imaging technologies have been developed to detect cathepsin and metalloproteinase activities, often using similar strategies as used in prodrug design 145. For instance, near-infrared fluorophores joined by peptide protease substrates become de-quenched upon cleavage 146. Protease cleavage can also lead to aggregation and signal enhancement of magnetic nanoparticles for MRI 147, and ^18^F PET tracers have been developed based on tight binding protease inhibitors 148. scRNAseq and imaging data underline the heterogeneity of protease activities at sites of disease across patients, particularly in solid cancers, due to variability in both the level of immune-cell infiltration, and in the regulated expression and activity of proteases in the cells that are present 37. This variability motivates companion diagnostic or theranostic approaches to identify patients likely to respond to protease-sensitive prodrugs or protease inhibitors.

**CYP family enzymes.** Some metabolic enzymes including cytochrome P450 oxidases are expressed in phagocytes, although they are more associated with processing endogenous lipid and sterol inflammatory mediators rather than xenobiotics. The cytochrome P450 oxidase CYP27A1, also known as sterol 27-hydroxylase, is expressed in MΦ and generates 27-hydroxycholesterol. This product is a selective estrogen receptor modulator and liver X receptor agonist, has been implicated in linking hypercholesterolemia with breast cancer development 149, and has consequently been considered as a adjuvant drug target in breast cancer. Cytochrome P450 2S1 (CYP2S1) is also expressed in MΦ, exhibits epoxygenase activity, and acts on fatty acids, prostaglandins, and vitamin D3 150.

**Figure 9:**
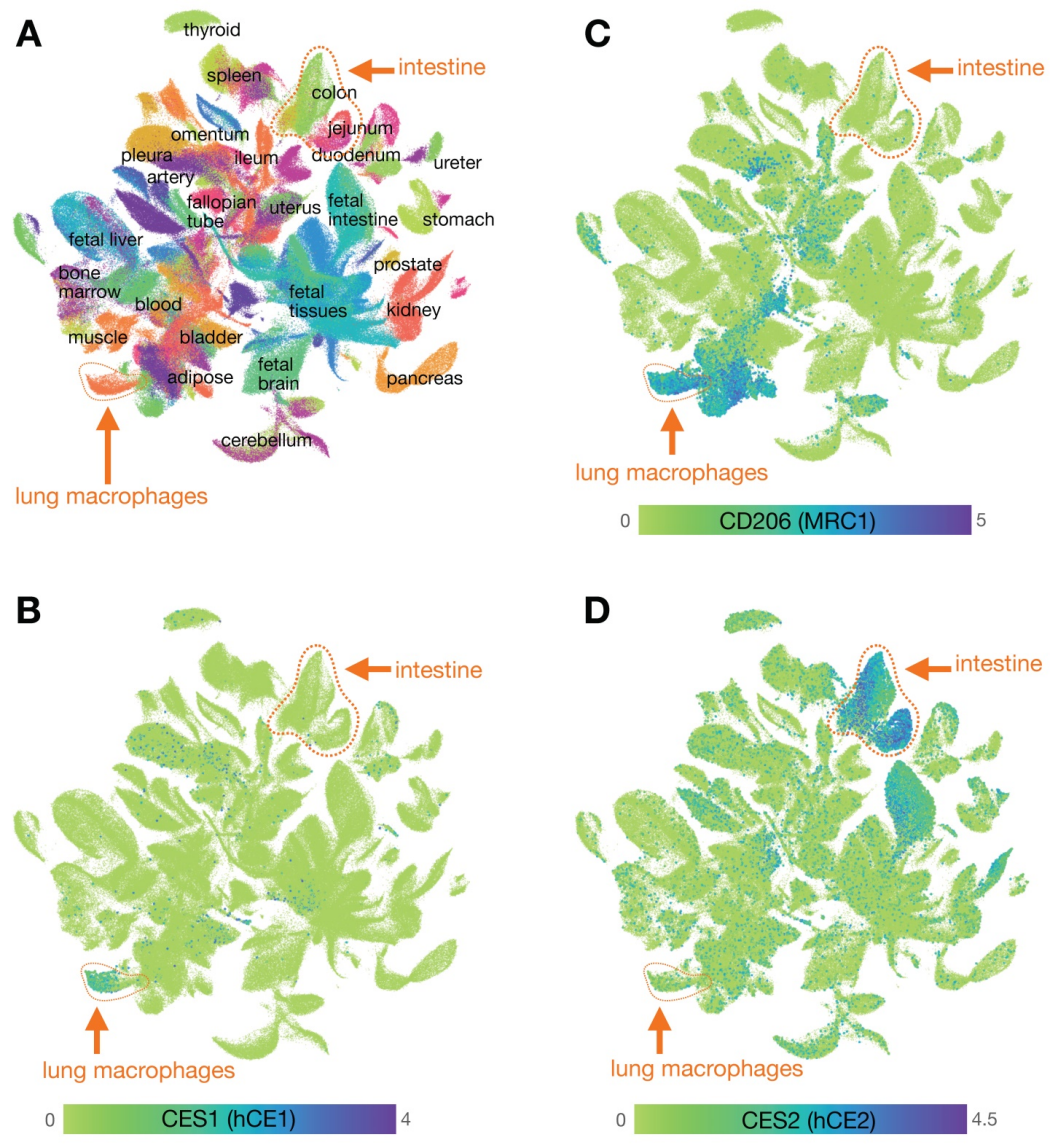
** Mapping carboxylesterase expression using the Human Cell Landscape. A)** Clustering of scRNAseq data from the human cell landscape project 29 shows >700,000 individual cells colored according to their tissue of origin. Epithelial cells from the intestine (primarily EpCAM+ cells including from the colon, rectum, jejunum, and duodenum) and myeloid cells from the lung (primarily CD68+ CD206+ cells, referred to here as lung MΦ) are highlighted. **B)** *CES1* is found expressed in the cluster of cells that includes high levels of lung MΦ, and is relatively absent in intestinal epithelium. **C)** Expression of CD206 is high in the cluster of *CES1*+ cells identified as lung MΦ, but is not restricted to that population. **D)** *CES2* is highly expressed in the intestinal epithelium compared to lung MΦ. See CellXGene and https://db.cngb.org/HCL/ for software to generate plots, described in 29.

**Carboxylesterase 1.** Carboxylesterase 1 (CES1, also known as human carboxylesterase 1, hCE1) can be highly expressed in phagocytes such as MΦ, and in the past CES1 has been referred to as monocyte esterase. CES1 is not specific to phagocytes, and the majority of carboxylesterase processing of xenobiotics occurs in the liver (by CES1) and intestine (by CES2) (**Fig. 9**). Nonetheless, in tissues such as the lung, phagocytes like alveolar MΦ express CES1 and can metabolize inhaled substances before they reach the liver 151. Relevant CES1 substrates include narcotics and organophosphate toxins such as sarin and VX gases.

CES1 can also activate prodrugs, including steroids, chemotherapeutics, and anti-virals like oseltamivir (marketed as Tamiflu) (**Fig. 8d-e**) 152. CES1 and CES2 have many shared substrates, but some prodrugs such as irinotecan show preferential cleavage by CES2, which is not typically expressed in phagocytes 153. In other cases, prodrugs can be encapsulated in long-circulating liposomes or other nanoparticles, which can be efficiently taken up at target sites of phagocyte accumulation before being metabolized in the liver. Liposomal irinotecan (Onivyde) is thought to rely on local uptake and prodrug activation by TAM, in part through MΦ-expressed CES1 9. Onivyde response in patients was correlated with the ability of tumors to accumulate ferumoxytol, supporting the model that MΦ contribute to delivery and activation of liposomal irinotecan in tumors 9. Esterase-activated steroid prodrugs have also been developed for encapsulation within lipid nanoparticles, in one example to mitigate unwanted immunogenic effects of siRNA delivery 154. The steroid ciclesonide is nebulized as a treatment for asthma and allergic rhinitis, and may be activated directly in the lung by CES-expressing epithelium and phagocytes 155.

Even orally administered prodrugs may be partly activated by phagocytes at sites of disease. For instance, animal models have shown higher active metabolite concentrations of the CES1 substrate oseltamivir in the lung than in circulation 156, suggesting further metabolic activation in tissue, despite most being systemically activated. Systemically administered prodrugs have been developed to mitigate exposure of active compound outside of targeted cell populations, for instance with the CES1 substrate and aminopeptidase inhibitor tosedostat, which has been developed for the treatment of myeloid leukemias. Tosedostat is one of a class of drugs designed with an “esterase sensitive motif” approach that in principle targets CES1-expressing monocytes, MΦ, and leukemias that arise from phagocyte myeloid precursors. Unfortunately, studies with tosedostat revealed that drug resistance emerged as leukemia cells adapted to lose expression of CES1 157.

Achieving local CES1-mediated prodrug activation requires balancing on-target activation with metabolization by other esterases in circulation such as serum paraoxonase/arylesterase 1 (PON1), which is also known as A esterase. In fact, PON1 activity has been used to design so-called “soft drugs” that achieve local activity by becoming metabolized to inactive forms upon reaching circulation 158. Complicating these efforts, carboxylesterases are distinct between mice and humans, and consist of 20 and 6 members, respectively. Mouse carboxylesterases are not highly expressed in MΦ 37, 159. In contrast, mouse and rat carboxylesterase Ces1c is present at high levels in plasma and has been noted to degrade antibody-drug conjugates faster than the rate seen in humans, motivating use of Ces1c^-/-^ mice to better model PK of antibody drug conjugates 160. The same approach has been applied for the antiviral remdesivir, which shows efficacy against the SARS-CoV-2 coronavirus.

## Phagocytes as drug depots

Nanoparticles and antibodies have both been used to more safely deliver toxic drugs to target cell populations in multiple diseases, for instance with PEGylated liposomal doxorubicin (Doxil) used to treat ovarian cancer, Kaposi's Sarcoma, and multiple myeloma, and the ADC ado-trastuzumab emtansine (Kadcyla) used to treat HER2+ metastatic breast cancer. ADCs and drug-loaded nanotherapies in oncology can have high off-target accumulation in phagocytes 81, 161, 162. The functional consequence of this uptake depends on the drug formulation and the biology of the phagocytes in which they accumulate. TAM can serve as drug depots that redistribute cytotoxic payload to surrounding tumor cells after they have taken up the ADC or nanoparticle delivery vehicle, leading to desired bystander killing 7, 15, 163. In follow-up, MΦ depletion decreased intratumoral accumulation of therapeutic nanoparticles and resulted in tumor growth 7. MΦ depletion using clodronate liposomes similarly reduced the effectiveness of tumor-targeted ADC, as did the use of ADC mutants with attenuated FcγR1 binding 163. TAM-mediated bystander killing has also been shown in mouse models using TAM-targeted aptamers that indirectly deliver the chemotherapy doxorubicin to tumor cells 164. In addition to the cytotoxic payload, TAM can release nanoparticles themselves after initial uptake, thus promoting subsequent delivery to neighboring tumor cells 165. The uptake of nanoparticles by phagocytes has also been used to affect neighboring cells by stimulating phagocyte production of cytokines 93, chemoattractants 166-167, or vaccine-targeted antigens 168. In other cases, phagocyte uptake may lead to unwanted drug sequestration or systemic clearance, and studies have shown how depleting MΦ 162, or saturating their ability to take up materials such as nanoparticles 169-171, can improve on-target delivery. MΦ-targeting agents such as the dextran-based nanoparticles Macrin and ferumoxytol, therefore, have potential utility as companion diagnostics for agents that are affected by drug depot, myeloid-reprogramming, or sequestration effects 15, 21, 169.

## Exploiting myeloid-shaped tissue environments for drug delivery

**Figure 10:**
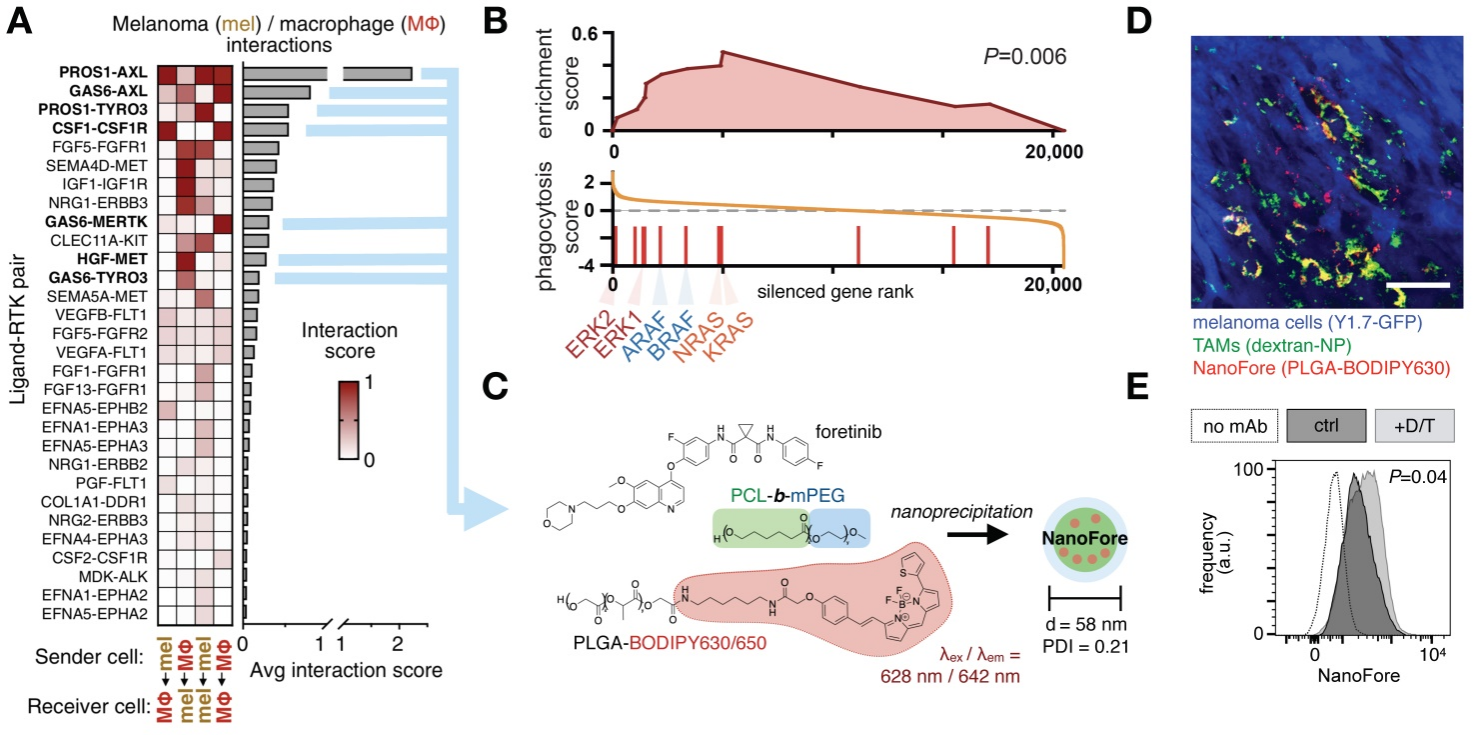
** Clinical kinase inhibitors influence tumor associated MΦ to improve nanotherapy delivery. A)** scRNAseq from BRAF-mutant melanoma biopsies tabulated ligand-receptor communication between malignant melanoma cells (“mel”) and MΦ across >1100 known ligand-receptor pairs; top receptor tyrosine kinase (RTK) pathways are shown. **B)** Gene set enrichment analysis of a genomic CRISPR screen shows that genetic silencing of MAPK/ERK pathway components — including RAS, RAF, and ERK proteins — leads to enhanced uptake of nanoparticles in MΦ. Genes were ranked according to the effect their CRISPR-mediated silencing had on nanoparticle uptake. MAPK/ERK components were enriched in increasing uptake.** C)** A nanoformulation encapsulates the multi-kinase inhibitor foretinib into micelles composed of poly(ε-caprolactone)-block-methoxy poly(ethylene glycol) (PCL-*b*-mPEG) and poly(lactic-*co*-glycolic) acid (PLGA), producing “NanoFore.” Key drug targets are boldfaced in *A*. **D)** NanoFore accumulates in tumor associated MΦ within melanoma allografts, as measured by confocal microscopy. Macrin (dextran-NP; see Fig. 2) was used to label MΦ. Scale bar, 50 μm. **E-F)** As predicted by the CRISPR screen in *B*, inhibition of MAPK/ERK activity with clinically-used BRAF inhibitor (dabrafenib) and MEK1/2 inhibitor (trametinib) “D/T” led to enrichment of MΦ in tumors (see ref. 11), and a roughly 2x increase in NanoFore uptake in MΦ on a per-cell basis, as measured by flow cytometry. For all, adapted with permission from 11, copyright 2020, and with data from 200.

Drug pharmacokinetics are influenced by abnormal vascular structures and permeability, interstitial fluid pressure change, lymphatic function, and extracellular matrix composition. These issues are particularly acute in solid cancers and with nanoparticles, which are thought to passively accumulate in some solid tumors via the EPR effect 4, 5, 172. Myeloid cells play central roles in shaping the vasculature, extracellular matrix, and overall EPR effect of tissues. For instance, perivascular TAM and neutrophils can produce growth factors including vascular endothelial growth factor (VEGF) to promote angiogenesis and metastasis in solid tumors 173, and this also can promote local bursts of vessel permeability and drug penetration into tissue 8, 174. As an example of how this may be exploited to enhance drug delivery in tumors, low-dose radiation was used to enrich solid tumors for MΦ content, which led to altered tumor vascularization and improved nanotherapy delivery in a MΦ-dependent manner 8, 170. Targeted kinase inhibition, immunogenic chemotherapy, and immune checkpoint blockade have all been shown to stimulate myeloid infiltration and subsequent nanoparticle accumulation in tumors 15, 175, 176. In the case of targeted mitogen activated protein kinase (MAPK) - extracellular related kinase (ERK) inhibition in Braf^V600E^ cancers, inhibition of the MAPK/ERK pathway using a clinical MEK1/2 inhibitor enriched xenografts for TAM content, stimulated VEGF and growth factor production in MΦ, stimulated MΦ to more avidly accumulate nanoparticles, and therefore led to greater nanoparticle uptake in treated tumors (**Fig. 10**) 11. Analysis of these tumor cell populations by scRNAseq elucidated the complex ligand-receptor communication that occurs between tumor cells and MΦ, offering new avenues for therapeutic intervention.

## Conclusions and Future Directions

Although this review outlines examples of MΦ imaging and phagocyte involvement in drug distribution and inactivation, the ability to intervene in these mechanisms to impact patient outcomes remains limited. One of the challenges remains the scope required to completely characterize the contribution of each immune cell type, while also maintaining the capacity to serially-sample and track the “kinetics” aspect of drug PK. In terms of multiplexing breadth at single-cell levels, scRNAseq is unmatched in the information provided: gene expression profiles can be used to subset cells, identify unique surface markers, and trace lineages. This information complements molecular imaging, as the unique cellular markers can be used to map cell subsets across time and space in a minimally-invasive manner, including across other types of immune cells such as T-cells 177-181. When used in this way a single gene, a signature, or even a cell type can become a biomarker to guide drug discovery efforts. As therapeutic modalities become increasingly complex in the age of cell and gene therapy, small molecule cocktails, and bi-specific engagers, the shift to more complex and comprehensive biomarkers may complement theranostic imaging of these agents 177,178,181. Multiplexed measurements like the recently approved FoundationOne CDX companion diagnostic for MET inhibition in non-small cell lung cancer (Foundation Medicine, Inc., Cambridge, MA) or the NanoString Prosigna Breast Cancer gene signature assay (NanoString Technologies, Seattle, WA) signal that transcriptomic signatures may someday be as commonplace as the longitudinal multidimensional diagnostic information provided by traditional radiographic medicine. Despite this promising future, hurdles remain.

Tissue heterogeneity makes sampling for transcriptomic analysis particularly difficult. Appreciation for heterogeneity is reflected in recent efforts to integrate spatial coordinates into single cell RNAseq and probe hybridization methods 182-184. Photoactivatable probes have even been used to guide the precise sampling of tissue for single cell analysis 185. T-cell receptor (TCR) sequencing is now a routine method that tracks the expansion of T cell clones over time 186, 187, and methods to monitor B cell diversity have been made commercially-available 188. Without a clonal marker like the TCR/BCR, MΦ and neutrophil expansion and turnover are still measured primarily with fluorophores detected by microscopy or flow cytometry 189. New methods to barcode monocytes for lineage tracing are currently in development 190. The integration of pooled CRISPR screens with scRNAseq may finally bring myeloid cells into the scope of functional genomics, permitting the testing of the influence of thousands of genes in parallel on monocyte differentiation and niche formation 31, 191.

Transcriptomic analysis has underlined that immune cells aren't held in static states, but are ever-changing in response to environmental cues. The artifacts introduced by tissue dissociation and cell/nuclei prep for transcriptional analysis 192 may be better understood and partially alleviated with complementary in situ imaging for cell stress hallmarks or population loss. Another way to counteract the tightly-regulated production and degradation of many inflammatory mRNAs is to focus instead on the epigenetic level. Chromosomal accessibility measured by ATACseq has provided a clearer picture of the differences between reversible and permanently-dysfunctional lymphocytes 193. Despite these insights, transcriptomics still provides just a snapshot in time, and the requirement for tissue sampling is greatly limited by the accessibility of clinically-relevant diseased tissues. Molecular imaging contextualizes the often transient transcriptomic information encoded in multiplexed single cell approaches. As this information is integrated, the hope is that new treatment biomarkers, resistance mechanisms, and cell-specific drug targets will be identified.

Given the broad involvement of macrophages in human disease and treatment response, particularly in inflammation, infection, and cancer, macrophage-centered diagnostics represent a practical application for transcriptomic and spatial technologies. One clear translational area lies in the development of prognostic or predictive biomarkers. Multiple clinical studies have correlated imaging and/or other readouts of macrophage behavior with disease progression, response to macrophage-influenced therapies such as liposomal irinotecan in advanced solid cancers 9, and response to PD1-targeted immune checkpoint blockade for instance in melanoma 10, as described above. Aside from abundance, altered TAM distribution in the tumor and neighboring tissue can be predictive of treatment response 201. Macrophage shape 94; 202 and transcriptional phenotype 203 also represent potential pathological parameters that can inform patient stratification or serve as companion markers of surrogate endpoints in clinical trials. Practical, logistic, and economic considerations all are challenges in successfully moving such applications from research into practice.

## Abbreviations

ADC: antibody drug conjugate
ADCC: antibody dependent cell-mediated cytotoxicity
BCR: b-cell receptor
DC: dendritic cell
EPR: enhanced permeability and retention
scRNAseq: single-cell RNA sequencing
MΦ: macrophage
mAb: monoclonal antibody
MAPK: mitogen activated protein kinase
MRI: magnetic resonance imaging
NK: natural killer
PET/CT: positron emission tomograph/X-ray computed tomography
PK: pharmacokinetic
TAM: tumor associated macrophage
TCR: t-cell receptor
